# A machine learning model for cancer screening in dogs using comprehensive circulating microRNA profiles

**DOI:** 10.1093/jvimsj/aalaf071

**Published:** 2026-01-31

**Authors:** Ruisa Nishida, Masashi Takahashi, Kaori Ide, Masashi Yuki, Shunsuke Noguchi, Yu Furusawa, Hiroaki Hojo, Sora Harako, Ririka Horikawa, Takuya Mizuno, Yasuyuki Momoi

**Affiliations:** Research and Development Division, ARKRAY, Inc., Kyoto 602-0008, Japan; Joint Faculty of Veterinary Medicine, Kagoshima University Veterinary Teaching Hospital, Kagoshima University, Kagoshima 890-0065, Japan; Laboratory of Veterinary Internal Medicine, Department of Veterinary Medicine, Tokyo University of Agriculture and Technology, Tokyo 183-8509, Japan; Yuki Animal Hospital, Aichi 455-0021, Japan; Japan Animal Referral Medical Center Osaka, Osaka 562-0036, Japan; Joint Faculty of Veterinary Medicine, Kagoshima University Veterinary Teaching Hospital, Kagoshima University, Kagoshima 890-0065, Japan; Research and Development Division, ARKRAY, Inc., Kyoto 602-0008, Japan; Research and Development Division, ARKRAY, Inc., Kyoto 602-0008, Japan; Research and Development Division, ARKRAY, Inc., Kyoto 602-0008, Japan; Laboratory of Molecular Diagnostics and Therapeutics, Joint Faculty of Veterinary Medicine, Yamaguchi University, Yamaguchi 753-8515, Japan; Division of Translational Research for One Medicine, Research Institute for Cell Design Medical Science, Yamaguchi University, Yamaguchi 753-8515, Japan; Japan Small Animal Cancer Center, Public Interest Incorporated Foundation Japan Small Animal Medical Center, Saitama 359-0023, Japan; Department of Veterinary Clinical Pathobiology, Graduate School of Agricultural and Life Sciences, The University of Tokyo, Tokyo 113-8657, Japan

**Keywords:** non-cording RNA, next-generation sequencing, oncology, liquid biopsy

## Abstract

**Background:**

MicroRNAs (miRNAs) are non-coding RNAs involved in cancer-related biological processes. To date, no studies have determined that liquid biopsy using miRNA can specifically identify dogs with cancer from a mixed population of dogs with and without non-malignant diseases.

**Hypothesis/Objectives:**

To assess the utility of a diagnostic model that differentiates dogs with cancer from a combined group of healthy dogs and dogs with non-malignant diseases, using miRNA profiles obtained by next-generation sequencing (NGS) and analyzed using machine learning.

**Animals:**

A total of 574 dogs were enrolled in the study: 168 with cancer, 138 with non-malignant diseases, and 268 healthy controls.

**Methods:**

Plasma samples from all dogs were analyzed by NGS to generate comprehensive miRNA profiles. Models were developed using DataRobot, based on the 50 most highly expressed miRNAs. The optimal model was selected based on area under the curve (AUC) results obtained using 5-fold cross-validation.

**Results:**

The miRNA-based model accurately distinguished dogs with cancer from those without cancer, achieving an AUC of 0.907, with both sensitivity and specificity of 0.85.

**Conclusions and clinical importance:**

A model integrating NGS-derived miRNA profiles with machine learning can serve as a diagnostic approach for cancer detection in dogs. Such a model can distinguish dogs with cancer from both healthy dogs and those with non-malignant disease. These findings suggest that such a model could be used as a screening test for dogs with cancer in veterinary practice.

## Introduction

Cancer is the leading cause of death in dogs.^1,2^ In veterinary medicine, interest in the application of liquid biopsy for cancer screening is becoming increasingly widespread. The primary goal of cancer screening tests is to identify those animals that should undergo more detailed diagnostic tests. Because numerous dogs presented to veterinary hospitals already have some form of illness,^3,4^ effective cancer screening tests must be capable of distinguishing dogs with cancer from those with other, non-malignant diseases. In particular, when cancer screening is incorporated into routine health care, dogs with common non-malignant conditions should be classified as negative. However, previous studies have compared dogs with cancer only to healthy dogs,^5,6^ and to date, no studies have been reported that explicitly included diseased dogs without cancer.

MicroRNAs (miRNAs) are a single-stranded, non-coding RNAs approximately 20 bases in length that have been implicated in various cancer-related processes, including promotion of proliferative signaling, evading growth suppressors, resisting cell death, facilitating invasion and metastasis, and inducing angiogenesis.^7^ In veterinary medicine, miRNAs also have attracted attention as potential biomarkers for disease.^8^ In a previous study, we demonstrated the potential application of miRNA-based diagnostic models as disease screening tests.^9^ However, no miRNA studies conducted to date have employed liquid biopsy samples to clarify whether miRNAs can differentiate between cancer and non-malignant diseases. Consequently, the potential of miRNAs for cancer screening in dogs remains unestablished.

Next-generation sequencing (NGS) allows for single-nucleotide resolution and is well-suited to generating comprehensive miRNA expression profiles. In human medicine, a recent study reported the development of a lung cancer screening model that combines NGS with machine learning, demonstrating its clinical utility.^10^ In contrast, no studies in veterinary medicine have yet demonstrated the clinical utility of a cancer screening model based on comprehensive miRNA profiles generated by NGS.

Therefore, our aim was to evaluate whether a model that integrates NGS-based blood miRNA profiling with machine learning can distinguish dogs with cancer from a combined group of healthy dogs and dogs with non-malignant diseases.

## Materials and methods

### Research subjects and ethics

Plasma specimens were collected between February 2024 and December 2024 from 6 veterinary institutions: the University of Tokyo Veterinary Medical Center, Kagoshima University Veterinary Teaching Hospital, Tokyo University of Agriculture and Technology Animal Medical Center, Yuki Animal Hospital, Animal Medical Technology, and the Japan Animal Referral Medical Center. The sample size for each group was determined with the goal of exceeding that of previous studies,^9^ and specimens were collected up to the maximum number possible within the study period. A total of 574 dogs of various ages and health statuses were included in the study. The cancer group consisted of 168 dogs diagnosed with cancer based on pathological examination or cytology. Most plasma specimens from this group were obtained at the initial veterinary visit, before the initiation of treatment. Some specimens were collected at follow-up visits, including samples from 21 dogs that were already receiving treatment. The non-cancer disease group included 138 dogs diagnosed with non-malignant diseases based on appropriate clinical examinations. Plasma specimens were collected at routine health evaluations, initial visits, and follow-up visits. Specimens obtained during health evaluations or initial visits were obtained before treatment, whereas those obtained during follow-up visits included samples from 25 dogs undergoing treatment. The healthy group consisted of 268 dogs that exhibited no abnormalities at the time of examination or at a follow-up assessment conducted 3 months later. Plasma specimens for this group were collected at health evaluations. All dogs were privately owned, and informed consent was obtained from all owners before participation in the study. Our study was conducted using residual specimens acquired from each dog during the course of clinical examination.

### Collection of blood samples and extraction of miRNAs from plasma

Blood specimens were collected in EDTA blood collection tubes (MiniCollect II EDTA-2 K; Sekisui Medical Co., Ltd., Tokyo, Japan), and plasma was separated by centrifugation at 2000 × *g* for 5 min at room temperature within 2 h of collection. After centrifugation, plasma samples were transferred to microtubes containing a nucleic acid preservative. Plasma samples exhibiting hemolysis after centrifugation were excluded. All plasma samples then were stored at −80°C until RNA extraction. Total RNA, including miRNA, was extracted from plasma using a commercial RNA extraction kit (Maxwell RSC miRNA from Tissue or Plasma and Serum Kits; Promega Corporation, Madison, WI, USA). Each RNA extract then was aliquoted into 2 samples and stored at −80°C until library preparation for NGS.

### NGS library preparation and sequencing

Extracted RNA samples were processed for NGS analysis and libraries were prepared using an automated liquid handling system (Agilent Bravo NGS; Agilent Technologies Inc., Santa Clara, CA, USA). Library construction followed protocols established in a previous study.^11^ The resulting NGS library was pooled to a uniform concentration after quantification by automated electrophoresis (TapeStation System High Sensitivity D1000; Agilent Technologies Inc.). Sequencing then was performed on the pooled library using the NGS system (Ion S5 System; Thermo Fisher Scientific Inc., Waltham, MA, USA).

### Preparation of the dataset

Sequencing read files were processed by first trimming adapter sequences using the *cutadapt* tool.^12^ The resulting reads then were annotated against *Canis lupus familiaris* miRNA sequences from miRBase v22.1 using the *miraligner* tool.^13^ For each sample, raw read counts were computed by summing the reads corresponding to identical miRNAs. These miRNA read counts were normalized to reads per million (RPM) for each sample and subsequently log_2_-transformed for downstream analysis.

### Construction of the screening model

The screening model was developed using the AutoML platform DataRobot (DataRobot, Inc., Boston, MA, USA),^14,15^ which automatically generates and integrates over 60 classification models. Input data consisted of expression data from the 50 most highly expressed miRNAs. Model performance was assessed by 5-fold cross-validation, and the model yielding the highest area under the curve (AUC) was selected for evaluation. Candidate algorithms for the miRNA models included linear models (Elastic-Net, logistic regression, and stochastic gradient descent), tree-based methods (Random Forest, ExtraTrees, XGBoost, and LightGBM), support vector machines, neural networks (various Keras architectures), generalized additive models (GAM, RuleFit, and Eureqa), and simple baselines (eg, Majority Class Classifier).

### Statistical analysis

Principal component analysis (PCA), correlation matrices, boxplots, AUC values, and receiver operating characteristic (ROC) curves were generated using the R statistical analysis software package version 4.1.1.^16^ Principal component analysis was conducted using the *prcomp* function of the *stats* package and visualized with the *ggplot2* package. The coefficient of determination (*R*^2^) for the standard RNA data was calculated using the *cor* function of the *stats* package and visualized using the *corrplot* package. The AUCs and 95% CI were calculated using the *pROC* package. Welch’s *t*-test was applied to continuous variables, and χ-squared tests were applied to categorical variables to evaluate differences in patient characteristics and diagnostic performance. Statistical significance was defined as *P* < .05.

## Results

### Patient characteristics

A total of 574 dogs were included in the study, consisting of 268 healthy dogs, 138 dogs with non-malignant disease, and 168 dogs with cancer.

Table 1 shows the characteristics of each group (ie, healthy, non-malignant, and cancer groups) by age, sex, and breed. The median ages were 7.5, 11.0, and 12.0 years in the healthy, non-malignant disease, and cancer groups, respectively. A comparison of age between the 2 groups (healthy + non-malignant disease vs. cancer) showed a significant difference (*P* < .01). The study population included 74 intact males, 231 neutered males, 42 intact females, and 227 spayed females. A comparison of sex distribution between the same 2 groups showed a significantly higher proportion of males in the cancer group (*P* = .03). The most commonly represented breeds included poodles (*n* = 103), mixed-breed dogs (*n* = 90), dachshunds (*n* = 72), and chihuahuas (*n* = 54). Dogs in the cancer group were diagnosed with various malignancies (Table 2).^17^ Similarly, dogs in the non-malignant disease group were diagnosed with a range of conditions (Table 3).

**Table 1:** Demographic and clinical characteristics of the dogs included in this study.

		**Healthy (*n* = 268)**	**Non-cancer disease (*n* = 138)**	**Cancer (*n* = 168)**	***P*-value (healthy + non-cancer disease vs. cancer)**
**Age, months**	Median	90	132	144	<.01
	Range	6-193	10-219	19-207	
**Sex**	Male	141 (52.6%)	67 (48.6%)	97 (57.7%)	.03
	Intact	36	15	23	
	Neutered	105	52	74	
	Female	127 (47.4%)	71 (51.4%)	71 (42.3%)	
	Intact	26	9	7	
	Spayed	101	62	64	
**Breed**	Mixed breed	55	15	20	
	Poodle	46	27	30	
	Dachshund	20	23	29	
	Chihuahua	34	10	10	
	Shiba	20	8	8	
	Miniature schnauzer	12	3	10	
	Yorkshire terrier	12	9	4	
	Pomeranian	7	5	5	
	Maltese	7	2	1	
	Other	55	36	51	

**Table 2:** Cancer types included in the cancer group.

**Cancer classification**	**Number of specimens**	**Details**
**Melanoma**	22	Oral melanoma, subungual melanoma, cutaneous melanoma
**Lymphoma and lymphocytic leukemias**	21	Gastrointestinal lymphoma, multicentric lymphoma, chronic lymphocytic leukemia, cutaneous lymphoma, mediastinal lymphoma
**Malignant tumors of the respiratory system**	20	Nasal adenocarcinoma, nasal cavity chondrosarcoma, nasal cavity squamous cell carcinoma, nasal cavity transitional cell carcinoma, lung carcinoma, lung histiocytic sarcoma, nasal sarcoma
**Mast cell tumors**	17	Cutaneous mast cell tumor
**Tumors of the urinary system**	16	Bladder urothelial carcinoma, renal cell carcinoma
**Hemangiosarcoma**	9	Spleen hemangiosarcoma, subcutis hemangiosarcoma, cardiac hemangiosarcoma, bone hemangiosarcoma, hemangiosarcoma of unknown primary
**Perianal tumors**	8	Apocrine gland anal sac adenocarcinomas, Perianal Adenocarcinoma
**Malignant tumors of the male reproductive system**	7	Prostatic carcinoma, Seminoma
**Soft tissue sarcomas**	6	Soft tissue sarcoma
**Tumors of the endocrine system**	6	Insulinoma, thyroid carcinoma
**Tumors of the mammary gland**	6	Inflammatory mammary carcinoma, malignant mixed mammary tumor, mammary gland carcinoma
**Hepatobiliary tumors**	5	Hepatocellular carcinoma
**Oral tumors**	3	Oral squamous cell carcinoma
**Gastric cancer**	2	Gastrointestinal stromal tumor
**Myeloma-related disorders**	2	Multiple myeloma
**Tumors of the Nervous System**	2	Oligodendroglioma, gliosarcoma
**Intestinal tumors**	1	Colon adenocarcinoma
**Tumors of the female reproductive system**	1	Granulosa cell tumor
**Thymic carcinoma**	1	Thymic carcinoma
**Other Tumors**	13	Osteosarcoma, visceral sarcoma, salivary adenocarcinoma, chemodectoma, fibrosarcoma, malignant epithelial tumor, mucinous carcinoma, adenocarcinoma

Dogs within the cancer group in this study were assigned to 1 of 20 cancer types based on *Withrow and MacEwen’s Small Animal Clinical Oncology* (6th edition).

**Table 3:** Diseases included in the non-malignant disease group.

Non-cancer disease classification	Number of specimens
**Skin and subcutaneous diseases**	24
**Hepatobiliary diseases**	19
**Urological diseases**	17
**Cardiovascular diseases**	15
**Gastrointestinal diseases**	14
**Diseases of the nervous system**	13
**Endocrine diseases**	8
**Respiratory diseases**	8
**Immune-mediated diseases**	5
**Ocular diseases**	5
**Orthopedic diseases**	4
**Reproductive diseases**	1
**Other diseases**	5

Dogs within the non-malignant disease group were assigned to one of 13 disease types.

### Reproducibility of NGS analysis for the 50 most highly expressed miRNAs

**Figure 1:**
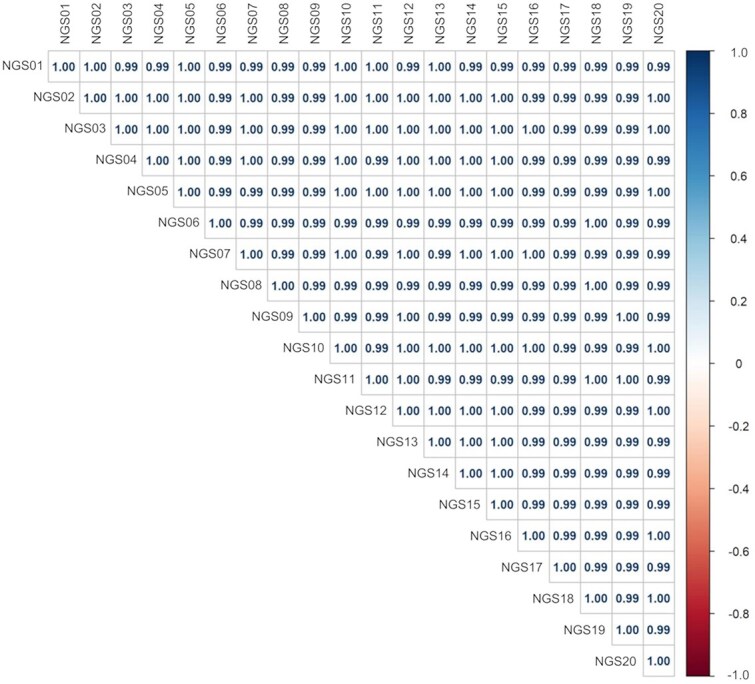
Pairwise correlation analysis of the 50 most highly expressed miRNAs across 20 measurements of a single pooled RNA sample. Twenty independent measurements were conducted, with correlation coefficients between samples represented by color-coded matrices.

**Figure 2:**
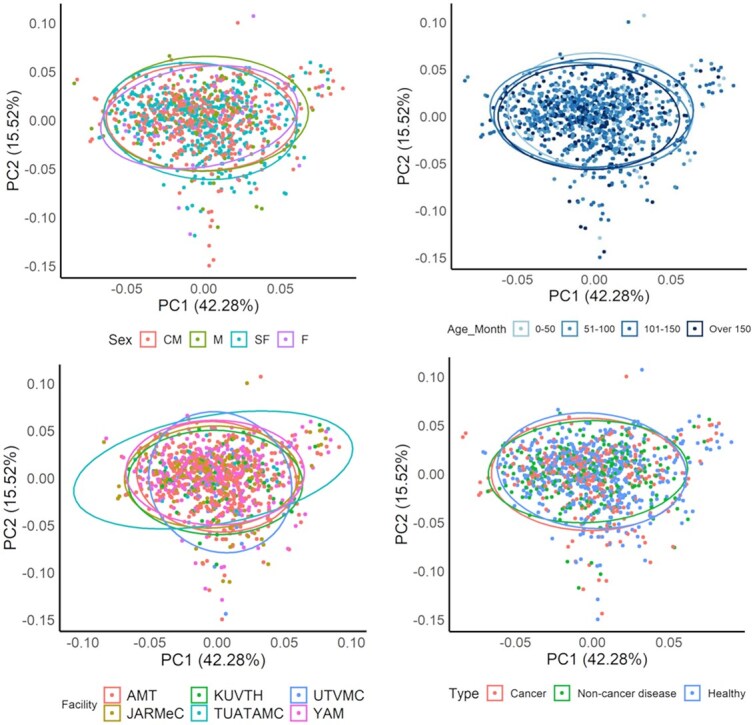
Principal component analysis (PCA) of the most highly expressed 50 microRNAs (miRNAs) across all samples. The PCA results are grouped by sex, age, facility, and type. Sex: CM = castrated male; F: Female; M: Male; SF: Spayed female. Facility: AMT: Animal Medical Technology; KUVTH: Kagoshima University Veterinary Teaching Hospital; UTVMC: University of Tokyo Veterinary Medical Center; JARMeC: Japan Animal Referral Medical Center; TUATAMC: Tokyo University of Agriculture and Technology Animal Medical Center; YAM: Yuki Animal Hospital.

Plasma specimens from 268 healthy dogs, 138 dogs with non-malignant diseases, and 168 dogs with cancer were analyzed by NGS to generate comprehensive miRNA expression profiles. To assess technical reproducibility, RNA extracted from the same plasma specimens was divided and analyzed in duplicate. In total, samples were processed across 20 independent NGS runs. In each sequencing run, standard RNA extracted from a single pooled plasma sample (Kitayama Labes Co., Ltd., Nagano, Japan) was included as a control. The correlation of miRNA expression values obtained from the standard RNA is shown in Figure 1. For all pairwise combinations of standard RNA replicates, the *R*^2^ for the log_2_-transformed RPM of the 50 most highly expressed miRNAs was ≥0.99, indicating high reproducibility. Principal component analysis of the 50 most highly expressed miRNAs was performed to visualize expression patterns across all samples (Figure 2). The samples did not form distinct clusters based on facility, sex, age, or diagnostic category.

### Selection of the highest-performing model (AUC = 0.907)

**Figure 3:**
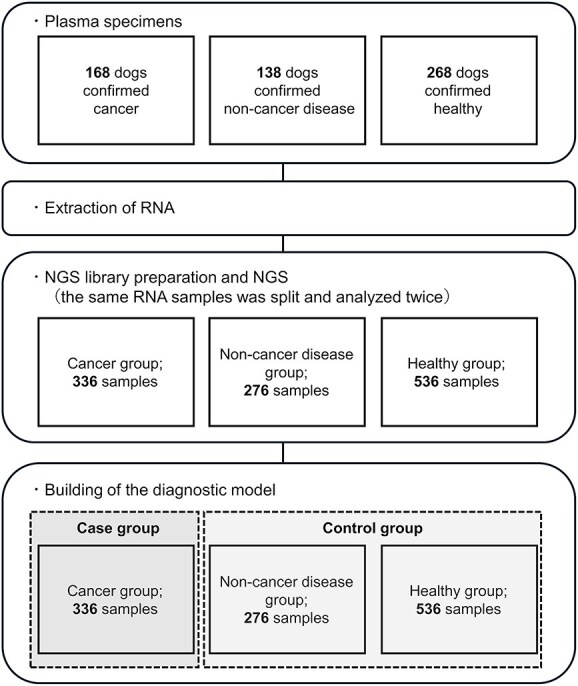
Workflow for the development of cancer screening models to distinguish dogs with cancer, dogs with non-malignant diseases, and healthy controls.

**Figure 4:**
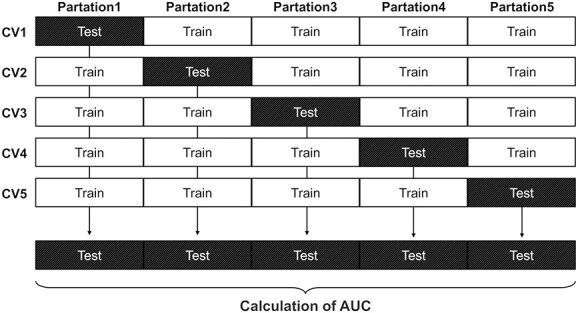
Illustration of 5-fold cross-validation and area under the curve (AUC) calculation.

**Figure 5:**
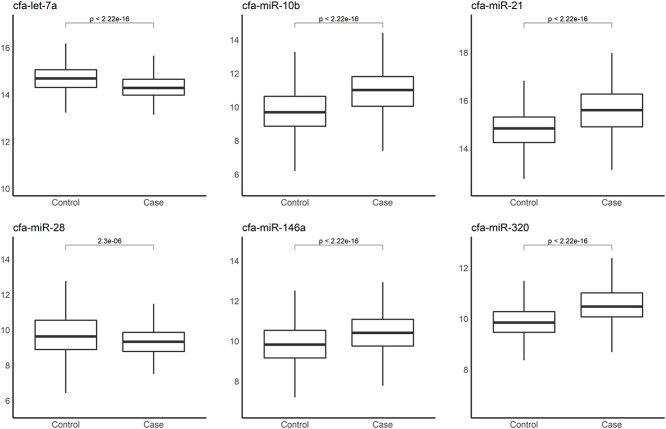
Box plots of the 6 miRNAs with the highest feature importance in the selected model, comparing expression levels between the control and case groups. The Y-axis shows log-transformed values of the normalized read counts for each miRNA.

**Figure 6:**
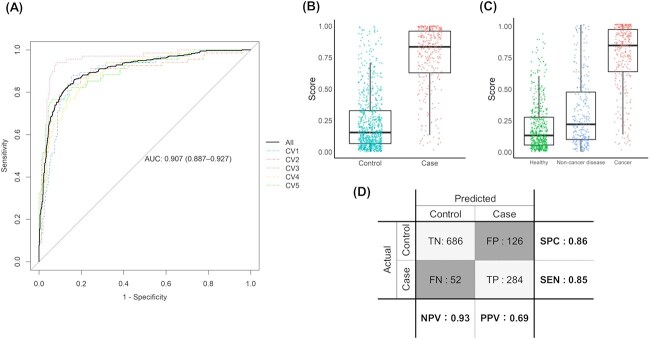
Model performance and validation metrics of the miRNA-based cancer screening model in dogs. (A) Receiver operating characteristic (ROC) curve. Dotted lines represent individual results from 5-fold cross-validation; solid line represents the aggregated ROC curve. (B, C) box-and-whisker plots of prediction scores generated by the model. (D) Model performance at the threshold of 0.48. Abbreviations: FN = false negative; FP = false positive; SEN = sensitivity; SPC = specificity; TN = true negative; TP = true positive; NPV = negative predictive value; PPV = positive predictive value.

The healthy + non-malignant disease group was designated as the control group, and the cancer group served as the case group (Figure 3). Binary classification models were constructed using the 50 most highly expressed miRNAs (Table 4). DataRobot generated 135 models, each evaluated using 5-fold cross-validation. All validation folds were pooled to calculate the overall AUC (Figure 4). For each fold in the cross-validation, paired data points derived from the same sample were assigned to the same partition to avoid data leakage. Among the 135 models, the Elastic-Net algorithm achieved the highest AUC and therefore was selected for further analysis. The expression levels of the miRNAs with high importance in this model are shown in Figure 5 for both the case and control groups. The selected model achieved an AUC of 0.907 (95% CI, 0.887-0.927). Using a threshold of 0.48, 284 of 336 cancer samples were classified as positive (sensitivity: 85%), whereas 686 of the 812 control samples were negative (specificity: 85%). When examined by subgroup, 207 of the 276 non-malignant disease samples tested negative (specificity: 75%), and 479 of 536 healthy samples tested negative (specificity: 89%; Figure 6). Notably, all cancer and non-cancer disease types were included in these analyses.

**Table 4:** Top 50 most highly expressed microRNAs (miRNAs).

cfa-let-7a	cfa-let-7b	cfa-let-7c	cfa-let-7f	cfa-let-7 g
cfa-miR-101	cfa-miR-103	cfa-miR-107	cfa-miR-10a	cfa-miR-10b
cfa-miR-122	cfa-miR-125a	cfa-miR-125b	cfa-miR-126	cfa-miR-128
cfa-miR-140	cfa-miR-142	cfa-miR-143	cfa-miR-144	cfa-miR-146a
cfa-miR-148a	cfa-miR-148b	cfa-miR-155	cfa-miR-15b	cfa-miR-16
cfa-miR-181a	cfa-miR-191	cfa-miR-192	cfa-miR-199	cfa-miR-21
cfa-miR-221	cfa-miR-223	cfa-miR-23a	cfa-miR-23b	cfa-miR-25
cfa-miR-26a	cfa-miR-26b	cfa-miR-28	cfa-miR-29a	cfa-miR-29c
cfa-miR-30d	cfa-miR-320	cfa-miR-423a	cfa-miR-425	cfa-miR-451
cfa-miR-486	cfa-miR-486-3p	cfa-miR-7	cfa-miR-92a	cfa-miR-93

**Figure 7:**
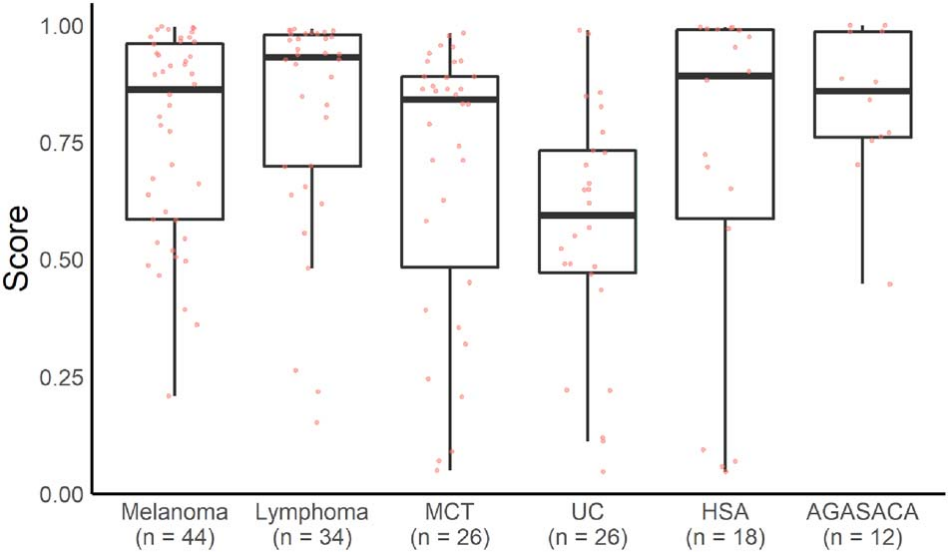
Sensitivity of the cancer screening model for each cancer type. Box-and-whisker plots showing prediction scores generated by the model for each cancer type. Cancer types: Melanoma (*n* = 44); lymphoma (*n* = 34); mast cell tumor (MCT; *n* = 26); urothelial carcinoma (UC; *n* = 26); hemangiosarcoma (HSA; *n* = 18), apocrine gland anal sac adenocarcinoma (AGASACA; *n* = 12).

**Figure 8:**
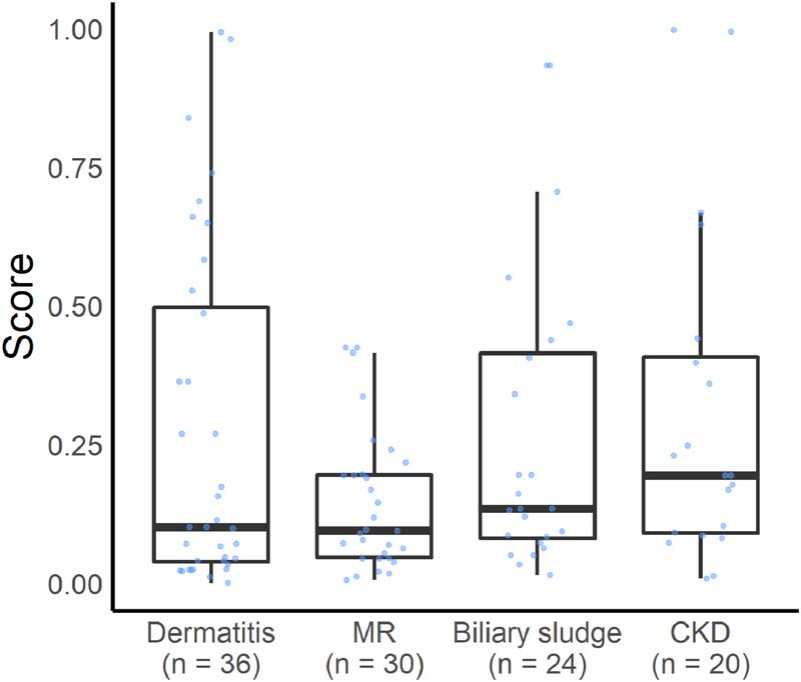
Specificity of the cancer screening model for each non-malignant disease type. Box-and-whisker plots show prediction scores generated by the model. Non-malignant disease types: Dermatitis (*n* = 36); mitral regurgitation (MR; *n* = 30); biliary sludge (*n* = 24); chronic kidney disease (CKD; *n* = 20).

For types of cancer and non-malignant diseases with relatively large numbers of samples, sensitivity and specificity were calculated for each type. At a threshold of 0.48, the sensitivity by cancer type was as follows: 90.9% for melanoma (*n* = 44), 91.2% for lymphoma (*n* = 34), 73.5% for mast cell tumor (*n* = 26), 73.1% for urothelial carcinoma (*n* = 26), 77.8% for hemangiosarcoma (*n* = 18), and 91.7% for apocrine gland anal sac adenocarcinoma (*n* = 12; Figure 7). Specificity by non-malignant disease was 72.2% for dermatitis (*n* = 36), 100% for mitral regurgitation (*n* = 30), 83.3% for biliary sludge (*n* = 24), and 90.0% for chronic kidney disease (*n* = 20; Figure 8).

## Discussion

We evaluated the potential of comprehensive blood miRNA profiling to differentiate dogs with cancer, healthy dogs, and dogs with non-malignant diseases. Principal component analysis of the 50 most highly expressed miRNA profiles did not indicate clear separation among the 3 groups. However, when combined with machine learning, the miRNA data facilitated accurate discrimination among these groups, yielding an AUC of 0.907.

The screening model achieved a specificity of 76% in the non-malignant disease group. One key factor contributing to the performance of the screening model is the composition of the non-malignant disease group. In a previous study, we developed a diagnostic model to differentiate between diseased and healthy states.^9^ In that study, non-malignant disease specimens were collected by the referring veterinary hospital, which resulted in a limited range of disease types being sampled. However, our current study included a broader range of diseases, particularly those frequently encountered at primary care veterinary hospitals, such as dermatologic diseases. This broader scope likely improved the model’s capacity to distinguish cancer from non-malignant diseases.

Another possible reason for the improvement in the model is the reduction in noise that could interfere with cancer classification. Inter-batch and inter-facility variation can hinder the reliability of liquid biopsy-based screening tests.^18^ In our study, batch-to-batch variation was monitored using a standardized control substance, and no substantial differences were observed, indicating high measurement reproducibility. Additionally, PCA showed that clustering was not affected by facility source, suggesting minimal site-specific bias.

Labelling errors in sample classification can decrease the accuracy of machine learning models. In machine learning, mislabeled training data can degrade overall model performance.^19^ To mitigate this issue, meticulous attention was paid to sample annotation (ie, healthy status was confirmed by follow-up assessments). These measures helped to minimize sources of noise in cancer classification, thereby enhancing model performance.

It is common for some animals presented to veterinary hospitals to have underlying health conditions. The screening model developed in our study can identify dogs at particularly high risk for cancer, even in those with other comorbidities, making it well-suited for implementation as a screening tool in clinical veterinary settings.

Our study included a diverse set of cancer types in model development, with the expectation that the model would capture common features of malignancy. However, certain cancer types, such as mast cell tumors and urothelial carcinomas, showed lower sensitivity. Given that circulating miRNAs vary by cancer type,^20-22^ the observed differences in sensitivity are likely attributable to cancer-specific expression patterns. Other contributing factors also may play a role. For example, in mast cell tumors, this difference may reflect the inclusion of relatively low-stage cases, because mast cell tumors often arise in the skin and are therefore more readily detected at an early stage.^23^ Although tumor stage information was not collected in our study, the need to obtain such data in future studies to validate this hypothesis is recognized. In urothelial carcinoma, it is highly likely that more miRNAs are released into urine than into blood.^24,25^ Thus, measuring urinary miRNAs may enable more specific discrimination, However, because we have no prior experience with measurement of urinary miRNAs, it remains unclear whether reproducibility is comparable to that of blood-derived miRNAs. To further substantiate these hypotheses, additional validation studies stratified by both tumor stage and sample type are warranted. These studies may elucidate whether the observed lower sensitivity in mast cell tumors and urothelial carcinomas can be improved by targeting higher-stage cases or by employing alternative sampling approaches.

As in the case of cancer, miRNA expression can be altered in non-malignant diseases,^26-28^ potentially complicating discriminating between cancer and non-cancerous conditions. This possibility may explain the lower specificity observed for disease samples compared with samples from healthy dogs. Future studies should focus on developing discriminative models with improved cancer specificity and on classifiers tailored to specific cancer types. These advances will require expansion of sample sizes for each cancer type and disease category and analysis of the unique expression profiles associated with them.

Our study had 2 main limitations. The first limitation is related to practical implementation. The study was conducted under a rigorous protocol and standardized implementation of this screening method in routine veterinary practice would require hospitals to adhere strictly to the established protocol or adopt a simplified version suitable for broader application. Additionally, the high cost of measurement technologies such as NGS presents a barrier to practical adoption, emphasizing the need for cost reduction. Furthermore, the model was evaluated only by cross-validation, without external validation. In the future, validation of our discrimination model in an independent cohort would be beneficial. Continued investigation into these aspects is warranted to support future implementation. A strategy for implementation involves piloting the model at selected facilities where adherence to the protocol is feasible. Broader deployment then could be considered. For cost effectiveness, because NGS is well-suited for high-throughput processing, expanding the scale of application may contribute to decreasing overall costs. The second limitation concerns potential bias in the study population. Specifically, the study cohort included both young healthy dogs and older dogs with cancer, introducing an age bias between case and control groups. However, PCA of the 50 most highly expressed miRNAs did not identify distinct clustering based on age or health status, suggesting minimal impact from this bias. Additionally, analysis of the relationship between age and miRNA expression levels indicated that none of the 50 miRNAs incorporated in the model was strongly correlated with age (Table S1). Accordingly, it was concluded that age bias is unlikely to influence the model. Nonetheless, future model development should incorporate more demographically balanced cohorts and consider age as a covariate to eliminate potential confounding effects. In general, more extensive and detailed clinical studies are needed to validate and refine the model. A major focus of follow-up research will be to verify the model’s capacity not only to identify cancer, but also to predict its type and location.

In conclusion, we demonstrated that a comprehensive miRNA profile generated using NGS, when combined with machine learning, can serve as a biomarker for cancer detection in dogs. Notably, our miRNA model was able to distinguish cancer from non-malignant disease and healthy states in dogs. These findings support the potential use of miRNA profiling as the basis for a cancer screening test in veterinary settings. Future work should focus on the clinical applicability, integration, and further evaluation of the model into routine diagnostic practice.

## Supplementary Material

aalaf071_suppl_tab_1

## Off-label antimicrobial declaration

Authors declare no off-label use of antimicrobials.

## Institutional animal care and use committee or other approval declaration

Authors declare no institutional animal care and use committee or other approval was needed because this study used residual specimens collected from each dog during clinically indicated testing, and written informed consent for the use of these specimens in research was obtained from the owners.

## Abbreviations

AGASACA: apocrine gland anal sac adenocarcinoma
AUC: area under the curve
CI: confidence interval
CKD: chronic kidney disease
FN: false negative
FP: false positive
GAM: generalized additive model
HSA: hemangiosarcoma
MCT: mast cell tumor
miRNA: microRNA
MR: mitral regurgitation
NGS: next-generation sequencing
NPV: negative predictive value
PCA: principal component analysis
PPV: positive predictive value
ROC: receiver operating characteristic
SEN: sensitivity
SPC: specificity
SVM: support vector machine
TN: true negative
TP: true positive
UC: urothelial carcinoma
