# Supplementary material for: A machine learning model for cancer screening in dogs using comprehensive circulating microRNA profiles
Source: J Vet Intern Med. 2026 Jan 31;40(1):aalaf071. doi: 10.1093/jvimsj/aalaf071 (PMC12859747; doi:10.1093/jvimsj/aalaf071)
Supplement: aalaf071_suppl_tab_1 [file aalaf071_suppl_tab_1.docx]

Supplemental Table 1. Coefficients of determination (R²) between each miRNA and age.

| **miRNA** | **Control** | **Case** |
| --- | --- | --- |
| cfa-let-7a | 2.96.E-05 | 1.69.E-03 |
| cfa-let-7b | 1.57.E-03 | 1.98.E-02 |
| cfa-let-7c | 2.76.E-04 | 1.39.E-02 |
| cfa-let-7f | 3.51.E-03 | 1.09.E-02 |
| cfa-let-7g | 4.61.E-03 | 1.91.E-03 |
| cfa-miR-101 | 2.20.E-03 | 3.02.E-02 |
| cfa-miR-103 | 1.08.E-02 | 4.66.E-03 |
| cfa-miR-107 | 2.31.E-02 | 1.61.E-03 |
| cfa-miR-10a | 5.29.E-05 | 9.56.E-03 |
| cfa-miR-10b | 1.80.E-04 | 7.41.E-03 |
| cfa-miR-122 | 4.37.E-02 | 2.92.E-02 |
| cfa-miR-125a | 1.49.E-03 | 6.72.E-03 |
| cfa-miR-125b | 3.37.E-04 | 1.50.E-02 |
| cfa-miR-126 | 4.82.E-04 | 2.07.E-04 |
| cfa-miR-128 | 2.73.E-05 | 1.69.E-03 |
| cfa-miR-140 | 2.91.E-02 | 1.36.E-02 |
| cfa-miR-142 | 2.11.E-02 | 2.60.E-03 |
| cfa-miR-143 | 1.46.E-03 | 5.07.E-03 |
| cfa-miR-144 | 2.77.E-02 | 1.70.E-03 |
| cfa-miR-146a | 1.39.E-02 | 1.26.E-02 |
| cfa-miR-148a | 1.50.E-04 | 1.78.E-02 |
| cfa-miR-148b | 1.73.E-03 | 4.49.E-04 |
| cfa-miR-155 | 1.03.E-01 | 1.13.E-03 |
| cfa-miR-15b | 1.11.E-02 | 1.50.E-02 |
| cfa-miR-16 | 1.06.E-02 | 4.98.E-03 |
| cfa-miR-181a | 3.47.E-02 | 9.51.E-03 |
| cfa-miR-191 | 2.79.E-04 | 6.00.E-03 |
| cfa-miR-192 | 5.75.E-03 | 1.92.E-02 |
| cfa-miR-199 | 4.59.E-02 | 1.05.E-02 |
| cfa-miR-21 | 2.23.E-05 | 1.78.E-02 |
| cfa-miR-221 | 6.43.E-04 | 3.87.E-02 |
| cfa-miR-223 | 1.21.E-03 | 1.14.E-02 |
| cfa-miR-23a | 1.92.E-03 | 2.99.E-02 |
| cfa-miR-23b | 1.78.E-02 | 3.36.E-02 |
| cfa-miR-25 | 1.15.E-02 | 3.29.E-02 |
| cfa-miR-26a | 1.61.E-02 | 5.61.E-05 |
| cfa-miR-26b | 2.91.E-04 | 1.57.E-03 |
| cfa-miR-28 | 1.55.E-03 | 8.74.E-03 |
| cfa-miR-29a | 5.50.E-03 | 3.63.E-02 |
| cfa-miR-29c | 1.99.E-02 | 9.31.E-02 |
| cfa-miR-30d | 1.21.E-03 | 1.05.E-02 |
| cfa-miR-320 | 2.06.E-03 | 1.90.E-02 |
| cfa-miR-423a | 2.48.E-03 | 3.93.E-02 |
| cfa-miR-425 | 7.23.E-04 | 1.04.E-04 |
| cfa-miR-451 | 2.13.E-02 | 1.97.E-03 |
| cfa-miR-486 | 3.28.E-03 | 1.92.E-03 |
| cfa-miR-486-3p | 2.35.E-03 | 2.73.E-03 |
| cfa-miR-7 | 3.30.E-02 | 4.27.E-03 |
| cfa-miR-92a | 9.90.E-04 | 1.04.E-02 |
| cfa-miR-93 | 1.44.E-02 | 2.13.E-03 |

To prevent spurious correlations arising from mixing groups, the R² were calculated separately for the control and case groups.
